# On the geometrically exact low-order modelling of a flexible beam: formulation and numerical tests

**DOI:** 10.1098/rspa.2018.0423

**Published:** 2018-08-08

**Authors:** C. Howcroft, R. G. Cook, S. A. Neild, M. H. Lowenberg, J. E. Cooper, E. B. Coetzee

**Affiliations:** 1 Department of Aerospace Engineering, University of Bristol, Queen's Building, University Walk, Bristol BS8 1TR, UK; 2 Future Projects Office, Airbus Operations Ltd, Pegasus House, Bristol BS34 7PA, UK

**Keywords:** reduced order, geometrically exact, nonlinear dynamics, shape function, Timoshenko beam, non-planar

## Abstract

This paper proposes a low-order geometrically exact flexible beam formulation based on the utilization of generic beam shape functions to approximate distributed kinematic properties of the deformed structure. The proposed *nonlinear beam shapes* approach is in contrast to the majority of geometrically nonlinear treatments in the literature in which element-based—and hence high-order—discretizations are adopted. The kinematic quantities approximated specifically pertain to shear and extensional gradients as well as local orientation parameters based on an arbitrary set of globally referenced attitude parameters. In developing the dynamic equations of motion, an Euler angle parametrization is selected as it is found to yield fast computational performance. The resulting dynamic formulation is closed using an example shape function set satisfying the single generic kinematic constraint. The formulation is demonstrated via its application to the modelling of a series of static and dynamic test cases of both simple and non-prismatic structures; the simulated results are verified using MSC Nastran and an element-based intrinsic beam formulation. Through these examples, it is shown that the nonlinear beam shapes approach is able to accurately capture the beam behaviour with a very minimal number of system states.

## 1. Introduction

Within the field of flexible structural modelling, one-dimensional beam models display a surprisingly wide applicability to the representation of numerous structural applications such as the modelling of aircraft wings, flexible satellites, turbine blades, communication towers, energy harvesters, atomic force microscopes and DNA molecules, to name but a few. Such treatments are typically suited to relatively slender structures with a single characteristic large dimension and, together with an appropriate constitutive material law, a one-dimensional representation may capture many aspects of the full three-dimensional problem. The same slender properties that make many physical structures amenable to beam representation often result in significant structural flexibility and, particularly for systems with free boundary conditions, this can lead to large deflections and subsequent geometric nonlinearity. Therefore, many studies treat such nonlinearity in kinematic frameworks permitting arbitrarily large rotation of either the structure directly, or to element frames in which structural sub-domains are treated. Of particular interest to the present study is the class of geometrically exact modelling methods first proposed in the seminal work of Reissner [1]. These formulations are centred around the concept of a local body fixed coordinate system in which the material deformation of the beam is cast. The kinematic description of the beam follows from knowledge of this local material deformation, as well as the span-varying orientation of the local body system, which may comprise a large rotation from the initial undeformed configuration; consequently, a global formulation of the flexible beam is attained in which geometric nonlinearity is inherently captured.

In a majority of cases, such geometrically nonlinear formulations are implemented using an element-based discretization of the structure. In [2,3], Simo and Vu-Quoc present a geometrically exact parametrization based upon the vector components of the global rotation and displacement from the initial undeformed configuration. This approach yields a geometrically nonlinear finite-element formulation of the flexible structure in terms of rotation and displacement states. Bauchau *et al.* [4] compare this formulation with the absolute nodal coordinate formulation of Shabana *et al.* [5] that uses gradient information rather than a rotational field in expressing the deflected geometry of the system. With a sufficiently fine discretization, linear strain–displacement laws may be used within each element frame. The class of co-rotational formalisms (first proposed in [6–8]) takes advantage of this by combining small local material deformation with arbitrarily large total rotations of the structure via the treatment of an element fixed (co-rotational) reference frame. Cesnik & Brown [9] allow for larger local deformations by accounting for the non-small linear variation of curvature within each element frame; the resulting formulation is cast directly in terms of curvature rather than displacement states. Consequently, the larger deformation allowed within each element frame affords the use of fewer elements in capturing geometric nonlinearity at the cost of increased complexity and bandwidth of the system matrices. Santos *et al.* [10] employ stress-resultant and displacement states in formulating a complimentary energy-based method for the representation of large static deformations. Together with additional displacement constraints, this allows for the treatment of nonlinearly deformed frame structures, free from the shear-locking phenomenon [11] and allowing for the accurate recovery of distributed stress. However, this comes at the cost of 24 degrees of freedom per element (see [12] for an overview of past research efforts and ongoing challenges in developing complementary energy principles for large deformation problems). Hodges [13] introduces the intrinsic beam formulation wherein constituent relations and the subsequent equations of motion are cast purely in a span-varying body-referenced intrinsic coordinate system. Patil *et al.* [14] use an element-based implementation of this formulation in studying the static aeroelastic response of a simple high-aspect ratio aircraft. Here, the intrinsic reference system effectively becomes the co-rotational system for each element and global geometric information is recovered via quaternion integration from root to tip. The review papers of Wasfy & Noor [15] and Shabana [16] further capture the large body of flexible multibody dynamic modelling literature.

A key feature of the above element-based descriptions is the generality with which structural configurations may be represented; however, this comes at the expense of a large number of system states. To reduce the problem size, one approach is to use model reduction methods; here one projects the full system onto a subset of modes using a regression analysis (for example, via implicit condensation [17,18] or enforced displacement [19] tests; see the review of Mignolet *et al.* [20] for further details) to fit the coefficients of higher-order terms, thus capturing nonlinear effects. However, the degree of nonlinear truncation required to render the regression problem tractable, as well as the inherent errors introduced when approximating the transient dynamics of the system, limits the domain of validity over which reduction techniques suitably approximate the full system.

For the current study, a different approach is adopted. Rather than reducing a high-order (large number of states) element-based model, the focus is cast instead on the low-order (minimal state) approximation of the full geometrically nonlinear dynamic problem. To this end, a *nonlinear beam shapes* approach is employed which approximates the kinematic quantities distributed along the full length of the flexible structure without subdivision into smaller elements. Compared to a typical element-based representation the reduction in system states can be significant. Such an approach is typical for the treatment of linear systems via, for example, Rayleigh Ritz, Galerkin and Modal type approximations (see standard texts on vibration modelling, e.g. [21]). In a geometrically nonlinear context, Patil & Althoff [22] consider higher-order shape functions as applied to the intrinsic beam formulation of [13]; here a set of shifted Legendre functions are used in approximating local angular velocity, translational velocity, force and moment quantities, expressed in the intrinsic reference frame. The resulting formulation is applied to the modelling of a flexible beam; however, a number of simplifying assumptions are required (i.e. treatment of a prismatic, constant property beam) in rendering the problem tractable to analysis. In the current paper, a geometrically exact flexible beam formulation is presented using a shape-based approximation of the kinematic beam quantities to yield a low-order continuous formulation of the problem. By using a global referencing attitude representation of the beam orientation at any spanwise location, analyticity is retained up to and including the integrands of positional quantities and their derivatives, thus lessening the computational requirements on the numerical integration of terms. The resulting dynamic equations are suitable for the representation of beam problems displaying generic span-varying geometric and material properties that typically exist in real structures; the underlying shape functions are kept general with candidate sets requiring only the satisfaction of a single kinematic constraint.

This paper is divided into two parts detailing firstly the modelling approach, followed by examples of its application. In §2, the mathematical formulation of the dynamic equations is presented, beginning with the variational statement of the geometrically exact problem and then introducing the chosen parametrization. The generic requirements and example application of an approximating shape basis is detailed in §3. In §4, a variety of validating test cases, which exercise various aspects integral to the treatment of a typical flexible beam problem, are presented. Throughout these test cases, additional results are generated as a baseline for comparison using both MSC Nastran (a well-established finite-element package capable of treating geometric nonlinearity) and the intrinsic beam formulation of [13] implemented as a finite-element code [23,24]. The latter modelling approach forms a particularly suitable base of comparison as, in addition to constituting a well-documented and validated nonlinear treatment, the resulting equations of motion take the form of a high-order system with fairly sparse system matrices and maximum order 2 nonlinearity. This serves as an interesting counterpart to the proposed formulation in this study which admits a greatly reduced number of states with fully populated form and a high degree of nonlinearity. Following the demonstration of the proposed nonlinear beam shapes approach in treating the discussed examples, conclusions are drawn in §5.

## 2. Presentation of dynamic equations

This section presents the derivation of the equations of motion describing a generic flexible nonlinear beam. This derivation proceeds with a geometrically exact beam treatment [2,3,25] wherein consideration of a local intrinsic coordinate system is used to capture the full geometric nonlinearity inherent in this class of flexible beam problem. By relating generic shape function sets to a spanwise-varying kinematic vector—consisting of three attitude, one extension and two shear parameters—a minimal state representation of the system is developed here which yields fast computational performance, easy truncation of shear and extensional deformations and admits representation as a nonlinear system of ordinary differential equations.

In §2a,b, a general statement of the geometrically exact variational problem is given wherein virtual work contributions are presented in terms of incremental displacement, shear, extensional and curvature deformations of the beam. In §2c, an Euler angle attitude representation is used to link these variational terms to a minimal set of shear, extension and rotational parameters. Following this, in §2d, a shape function discretization is used to split these parameters into spatial and temporal components yielding a minimal state representation of the problem in terms of the set ***q*** of time-dependent generalized states from which the equations of motion follow.

### (a) Kinematic description

**Figure 1.**
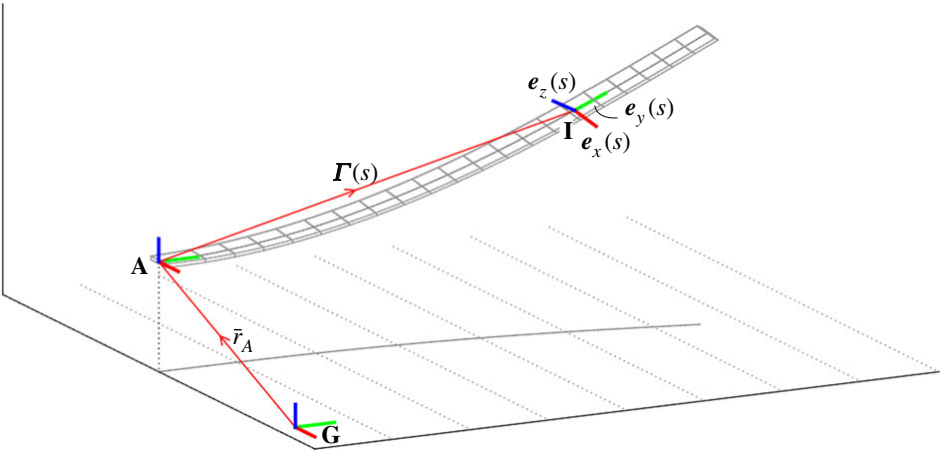
Reference coordinate systems and position vectors used in characterizing the three-dimensional beam geometry.

**Figure 2.**
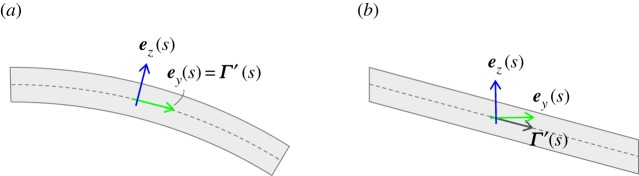
Illustration of the distinction between (*a*) bending and (*b*) shear-type structural deformation.

Consider first the flexible beam of length *L* depicted in figure 1, deemed to be a suitable representation of some slender three-dimensional structure, reduced about an arbitrary material reference line that forms the beam axis; the curvilinear coordinate *s*∈[0, *L*] is used to parametrize the distance along this axis. The axis itself is defined by the position vector ***Γ***(*s*) taken in some arbitrary reference system denoted **A** (here considered with origin placed at the beam root). This system is in turn embedded in the inertial reference system **G** with positional offset ${\bar{r}}_{A}$. Now take the intrinsic body-fixed system **I** (depicted at an example location *s* along the beam) represented by the orthonormal vector triad {***e***_*x*_, ***e***_*y*_, ***e***_*z*_}. In the undeformed configuration, ***e***_*y*_ lies parallel to the beam axis and ***e***_*x*_ and ***e***_*z*_ are mutually perpendicular directions in the cross-sectional plane normal to ***e***_*y*_. As the beam deflects the orientation of ***e***_*x*_ and ***e***_*z*_ (in the absence of cross-sectional warping) follow the evolution of their respective material lines while ***e***_*y*_ remains normal to the cross-sectional plane they span. In figure 2, example orientations of ***e***_*y*_, ***e***_*z*_ and ***Γ***′(*s*) (where the superscript •′ denotes differentiation with respect to *s*) are shown for the cases of planar pure bending (*a*) and pure shear (*b*). The difference in cross-sectional orientation for each sub-case illustrates the important point that, in the presence of shear deformation, ***e***_*y*_≠***Γ***′. Therefore, in general, the reference line ***Γ***′(*s*) comprises a spanwise integration along ***e***_*y*_ (including axial extension) in addition to shear deformations in the ***e***_*x*_ and ***e***_*z*_ directions. ***Γ***_[*G*]_(*s*) is thus given the form:

2.1 $$\Gamma_{\left[G\right]}(s)={\bar{r}}_{A[G]}+[R_{G,A}]\int_{0}^{s}(1+\varepsilon (\tilde{s}))\;e_{y[A]}(\tilde{s})+\tau_{x}(\tilde{s})\;e_{x[A]}(\tilde{s})+\tau_{z}(\tilde{s})\;e_{z[A]}(\tilde{s})\;d\tilde{s}$$

(cf. Reissner [1]), where *ε*(*s*), *τ*_*x*_(*s*) and *τ*_*z*_(*s*) denote axial extension, ***e***_*x*_ shear and ***e***_*z*_ shear deformations of the beam at each spanwise location, respectively. Each subscript in square brackets denotes the reference system (**G**, **A** or **I** depicted in figure 1) in which each vector quantity is cast. These coordinate systems are related by the rotation matrices [***R***_***G***, ***A***_] and [***R***_***A***, ***I***_] such that for any vector ***v***

2.2 $$v_{\left[G\right]}=[R_{G,A}]v_{\left[A\right]}=[R_{G,A}][R_{A,I}]v_{\left[I\right]}.$$

### (b) Virtual work terms

Following on from the kinematic description, the equations of motion describing the geometrically exact beam are now developed in weak form; individual terms are formulated via the principle of virtual work done by internal and external forces acting over incremental displacements, rotations and beam strains. These work terms are detailed in the following subsections and in each case, the variational quantities are related back to the intrinsic coordinate system depicted in figure 1.

#### (i) Material stress

In treating the stress terms of the system, strain deformations of the structural material are considered as lumped translational and rotational gradients along the parametric beam line ***Γ***(*s*). Hence the deformation vector ***ξ***(*s*) is introduced here which consists of the components

2.3 $$\xi (s)=(\tau_{x}(s),\varepsilon (s),\tau_{z}(s),\kappa_{x}(s),\kappa_{y}(s),\kappa_{z}{\left(s)\right)}^{T}.$$

Here, *κ*_*x*_(*s*), *κ*_*y*_(*s*) and *κ*_*z*_(*s*) give the ***e***_*x*_, ***e***_*y*_ and ***e***_*z*_ components of the beam curvature ***Γ***′′(*s*), respectively. The curvature may be written as^1^

2.4 $$\kappa ={\left(\kappa_{x},\kappa_{y},\kappa_{z}\right)}^{T}={\left(e_{y}^{\prime }\cdot e_{z},e_{z}^{\prime }\cdot e_{x},e_{x}^{\prime }\cdot e_{y}\right)}^{T}$$

often expressed in the compact form

2.5 $$\tilde{\kappa }=e^{T}e^{\prime },\;e=(e_{x}|e_{y}|e_{z}),$$

where $\tilde{\kappa }$ denotes the skew symmetric matrix formed from the vector ***κ*** = (*κ*_*x*_, *κ*_*y*_, *κ*_*z*_).

With these curvatures defined, the strain-induced internal force and moment distribution may be written as the six-component force/moment vector

2.6 $$F_{K}={\left(F_{Kx},F_{Ky},F_{Kz},M_{Kx},M_{Ky},M_{Kz}\right)}^{T}=-K\Delta \xi ,\;\Delta \xi =(\xi -\xi_{0}),$$

where ***ξ*_0_** denotes some minimal stress pre-curvature of the beam and the generic stiffness matrix is written as

2.7 $$K=\left(\begin{array}{cccc} K_{11}(s,\Delta \xi ) & K_{12}(s,\Delta \xi ) & \cdots & K_{16}(s,\Delta \xi )\\ K_{21}(s,\Delta \xi ) & K_{22}(s,\Delta \xi ) & \cdots & K_{26}(s,\Delta \xi )\\ \vdots & \vdots & \ddots & \vdots \\ K_{61}(s,\Delta \xi ) & K_{62}(s,\Delta \xi ) & \cdots & K_{66}(s,\Delta \xi ) \end{array}\right)\;K_{ij}=K_{j\;i}.$$

For the examples of this study, the linear stress–strain relationship is assumed wherein diag(***K***) = (*GA*, *EA*, *GA*, *EI*_*xx*_, *GJ*, *EI*_*zz*_) and all other entries are zero. Note however that, more generally, one may simply consider

2.8 $$F_{K}=F_{K}(s,\Delta \xi )$$

to be some arbitrary constitutive law relating the internal stress distribution to deformations **Δ*ξ*** about the beam reference axis ***Γ***(*s*) [25]. For structures with simple geometries and known material properties, this may be obtained analytically by relating the beam deformation vector ***ξ*** to the material strain tensor (e.g. [26]); for more complex structures, the constitutive relation may be obtained from physical or modelled tests of the full structure followed by the subsequent one-dimensional reduction about an arbitrary reference line [27]. In this general case of equation (2.8), the virtual work done over the beam, given the incremental strain deformation ***δξ***, is

2.9 $$\delta W_{K}=\int_{0}^{L}F_{K}(s,\Delta \xi )\cdot \delta \xi \;ds.$$

#### (ii) Structural damping

Formulation of the virtual work done by structural dissipation follows in much the same form as the preceding strain term. Considering again the variation ***δξ***, one may specify the function $F_{C}(\Delta \xi ,\dot{\xi })$ relating the internal structural damping force/moment ***F***_*C*_ to the strain rates $\dot{\xi }$. Thus, the virtual work for this incremental deformation takes the form

2.10 $$\delta W_{C}=\int_{0}^{L}F_{C}(s,\Delta \xi ,\dot{\xi })\cdot \delta \xi \;ds,\;\dot{\tilde{\kappa }}={\dot{e}}^{T}e^{\prime }+e^{T}{\dot{e}}^{\prime }.$$

#### (iii) Applied loads

*External forces.* The work done by an applied span-varying force vector ***F***_[*G*]_(*s*) with components in the global reference system is expressed with respect to the infinitesimal displacement of the reference line ***r***_*F*[*G*]_ along which it is applied. This reference line in the global system is given by

2.11 $$r_{F[G]}(s)=\Gamma_{\left[G\right]}(s)+a_{F}(s)e_{x[G]}(s)+c_{F}(s)e_{z[G]}(s).$$

Recall ${\bar{r}}_{A[G]}$ from figure 1 and define *a*_*F*_ and *c*_*F*_ as the offset of ***r***_*F*[*G*]_ from the beam reference line ***Γ*** in the ***e***_*x*_ and ***e***_*z*_ directions, respectively. This reference line follows the deformation of the beam structure and, in the absence of cross-sectional warping, *a*_*F*_ and *c*_*F*_ remain independent of the beam strain. Thus, the virtual work due to the applied load ***F***_[*G*]_(*s*) takes the form

2.12 $$\delta W_{F}=\int_{0}^{L}F_{\left[G\right]}(s)\cdot {\delta r}_{F[G]}(s)\;ds,$$

where the variational change in reference line is

2.13 $${\delta r}_{F[G]}(s)={\delta \Gamma }_{\left[G\right]}(s)+a_{F}(s)\delta e_{x[G]}(s)+c_{F}(s)\delta e_{z[G]}(s)$$

and

2.14 $$\begin{array}{rl} {\delta \Gamma }_{\left[G\right]}(s) & =\delta {\bar{r}}_{A[G]}+\int_{0}^{s}((1+\varepsilon (\tilde{s}))\;\delta e_{y[G]}(\tilde{s})+\delta \varepsilon (\tilde{s})e_{y[G]}(\tilde{s})+\tau_{x}(\tilde{s})\;\delta e_{x[G]}(\tilde{s})\\ & \;+\delta \tau_{x}(\tilde{s})\;e_{x[G]}(\tilde{s})+\tau_{z}(\tilde{s})\;\delta e_{z[G]}(\tilde{s})+\delta \tau_{z}(\tilde{s})\;e_{z[G]}(\tilde{s}))\;d\tilde{s}. \end{array}$$

Note that ***F***_[*G*]_ is general, in that its magnitude and orientation may vary arbitrarily in time and/or depend upon the states of the system, e.g. as in the case of a time-varying follower force.

*External moment*. Applied moments are denoted by the distributed vector ***M***_[*G*]_(*s*) for which the work done is considered with respect to the rotational variation ***δ𝜗***(*s*). This variation is written as

2.15 $${\delta \vartheta }_{\left[G\right]}(s)=\frac{1}{2}{\left(e_{x}(s)\times \delta e_{x}(s)+e_{y}(s)\times \delta e_{y}(s)+e_{z}(s)\times \delta e_{z}(s)\right)}_{\left[G\right]}$$

and thus

2.16 $$\delta W_{M}=\int_{0}^{L}M_{\left[G\right]}(s)\cdot {\delta \vartheta }_{\left[G\right]}(s)\;ds.$$

#### (iv) Kinetic terms

Consider the reference line ***r***_*m*_(*s*) intersecting the centre of mass of all infinitesimal cross sections along the length of the beam. The inertial terms are furnished by considering the work performed when imparting a change in the momentum of the beam over incremental displacements ***δr***_*m*_(*s*) and rotations ***δ𝜗***(*s*) of this reference line. The virtual work is thus written
2.17
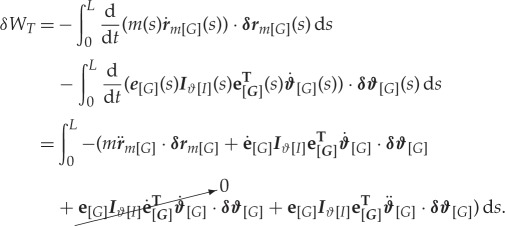
Expressions for ***r***_*m*_(*s*) and its variation *δ****r***_*m*_(*s*) take the identical form to equations (2.11) and (2.13) with the substitution $\left\{a_{F},c_{F}\}\to \{a_{m},c_{m}\right\}$. *m*(*s*) gives the mass per unit length along ***r***_*m*_(*s*) and ***I***_*𝜗*_(*s*) the sectional inertia matrix. Additional point masses may be incorporated via substitution of the appropriate Dirac delta representations onto the reference line ***r***_*m*_(*s*) and mass distribution *m*(*s*). The remaining accelerations $\ddot{\vartheta }$ and ${\ddot{r}}_{m}$ are detailed in the appendices.

### (c) Attitude representation

To further develop the describing equations of motion, one must relate the variational quantities ***δΓ***, ***δξ*** and ***δ𝜗*** to a consistent set of kinematic parameters over which the problem will be solved. Together these variations depend upon the deformation of the flexible beam as well as the translational and rotational motion of the reference system **A**.

For the deformation of the flexible structure, one may consider the components of the vector ***ξ*** = (*τ*_*x*_(*s*), *ε*(*s*), *τ*_*z*_(*s*), *κ*_*x*_(*s*), *κ*_*y*_(*s*), *κ*_*z*_(*s*))^T^ in the material frame as a natural description of this deformation. However, although the above variations are uniquely determined by these components, there is no closed-form solution directly relating the curvature in the material frame to ***𝜗***_[*A*]_ and ***Γ***_[*A*]_ for all but the simplest *κ*_*x*_(*s*), *κ*_*y*_(*s*), *κ*_*z*_(*s*) distributions (note that Cesnik & Brown circumvent this issue by assuming simplified *κ* approximations within individual element frames, building up complexity via discretization [9]).

**Figure 3.**
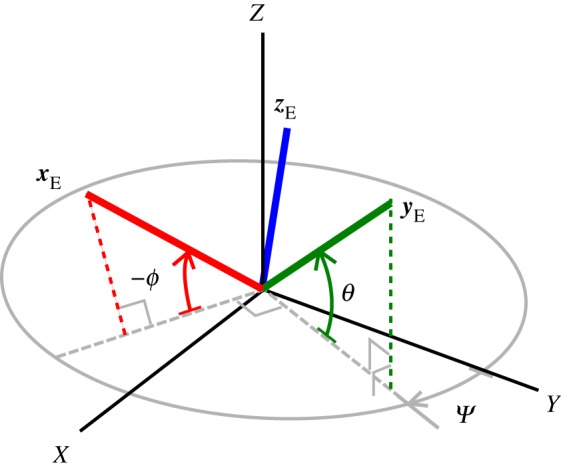
The set of Euler angles used to parametrize the intrinsic system orientation.

For the continuous formulation developed here, this lack of a closed-form relation complicates the treatment of these quantities and their derivatives. To address this issue, one notes from equations (2.1), (2.15), (2.3) and (2.4) that these variations (in the reference system **A**) are dependent on the intrinsic coordinate system **e**_[*A*]_ such that:

2.18 $$\begin{array}{rl} \Gamma_{\left[A\right]}(s) & =\Gamma_{\left[A\right]}(\tau_{x}(s),\varepsilon (s),\tau_{z}(s),e_{\left[A\right]}(p)),\\ \vartheta_{\left[A\right]}(s) & =\vartheta_{\left[A\right]}(e_{\left[A\right]}(p))\\ \;\mathrm{and}\;\xi_{\left[A\right]}(s) & =\xi_{\left[A\right]}(\tau_{x}(s),\varepsilon (s),\tau_{z}(s),e_{\left[A\right]}(p),e_{\left[A\right]}^{\prime }(p)), \end{array}\}$$

where ***p*** denotes the set of attitude parameters uniquely defining **e**_[*A*]_. There are numerous potential choices for such a parametrization including, for example, quaternions, rotation vector, Rodrigues parameters; these and other such parametrizations are detailed in [28]. For the remainder of this study, an example set of asymmetric 3-1-2 Euler angles is employed; like other three-parameter descriptions this has the advantage of a minimal state representation and in the context of the current formulation is found to yield fast computational performance. The attitude states—here denoted ***p*** = (*θ*, *ψ*, *ϕ*)—relating to this parametrization are depicted in figure 3. The indicated angular deflections are labelled in a consistent manner with the particular positive convention employed in equation (2.19). Given these angular definitions, and taking the reference system **A**,

2.19 $$\begin{array}{rl} e_{\left[A\right]} & =(e_{x[A]}|e_{y[A]}|e_{z[A]})=R_{E}(x_{E}|y_{E}|z_{E})R_{E}^{T}\\ & =R_{E}\left(\begin{array}{ccc} \;c_{s}s_{n}\psi \;c_{s}s_{n}\phi +s_{n}\theta s_{n}\psi s_{n}\phi & \;c_{s}s_{n}\theta s_{n}\psi & \;c_{s}s_{n}\psi s_{n}\phi -s_{n}\theta s_{n}\psi \;c_{s}s_{n}\phi \\ -s_{n}\psi \;c_{s}s_{n}\phi +s_{n}\theta \;c_{s}s_{n}\psi s_{n}\phi & \;c_{s}s_{n}\theta \;c_{s}s_{n}\psi & -s_{n}\psi s_{n}\phi -s_{n}\theta \;c_{s}s_{n}\psi \;c_{s}s_{n}\phi \\ -\;c_{s}s_{n}\theta s_{n}\phi & s_{n}\theta & \;c_{s}s_{n}\theta \;c_{s}s_{n}\phi \end{array}\right)R_{E}^{T}, \end{array}$$

where the shorthand convention *s*_*n*_ and *c*_*s*_ for the trigonometric operations ‘sin()’ and ‘cos()’ is used; ***R***_E_ denotes the optional transform specifying the mapping from the Euler axis (***x***_E_, ***y***_E_, ***z***_E_) to the intrinsic system (***e***_*x*_, ***e***_*y*_, ***e***_*z*_). Via the choice of ***R***_E_ one may place the *θ* =  ± 90° Euler singularities of the problem at generic opposing orientations of the intrinsic reference frame **A**. For example, when ***R***_E_ takes the value of the 3 × 3 identity matrix, the Euler singularities fall at intrinsic frame orientations satisfying the equality ***e***_*y*[*A*]_ = (0, ± 1, 0)^T^.

Note that an additional attitude and position parametrization can be assigned to the motion of system *A* with respect to the global reference **G** for problems where the root kinematics are not prescribed. This is useful in treating free problems, e.g. the modelling of a flexible aircraft in free flight [9,14,24,29]. For brevity, these terms are not furnished in this derivation but are easily appended to the problem; therefore, from here ${\bar{r}}_{A}(t)$ and [***R***_***G***, ***A***_](*t*) take the form of prescribed time-varying functions.

Given the aforementioned attitude parametrization, the following quantities may now be related directly to the Euler angles *θ*, *ψ* and *ϕ*.

*Curvature*:

2.20 $$\left(\begin{array}{c} \kappa_{x}\\ \kappa_{y}\\ \kappa_{z} \end{array}\right)=R_{E}\left(\begin{array}{c} \psi^{\prime }\;c_{s}s_{n}\theta s_{n}\phi +\theta^{\prime }\;c_{s}s_{n}\phi \\ \phi^{\prime }-\psi^{\prime }s_{n}\theta \\ -\psi^{\prime }\;c_{s}s_{n}\theta \;c_{s}s_{n}\phi +\theta^{\prime }s_{n}\phi \end{array}\right).$$

*Spin*:

2.21 $${\delta \vartheta }_{\left[A\right]}=R_{E}\left(\delta \theta \left(\begin{array}{c} \;c_{s}s_{n}\psi \\ -s_{n}\psi \\ 0 \end{array}\right)+\delta \psi \left(\begin{array}{c} 0\\ 0\\ -1 \end{array}\right)+\delta \phi y_{E}\right)$$

and

2.22 $${\ddot{\vartheta }}_{\left[A\right]}=R_{E}\left(\ddot{\theta }\left(\begin{array}{c} \;c_{s}s_{n}\psi \\ -s_{n}\psi \\ 0 \end{array}\right)+\dot{\theta }\dot{\psi }\left(\begin{array}{c} -s_{n}\psi \\ -\;c_{s}s_{n}\psi \\ 0 \end{array}\right)+\ddot{\psi }\left(\begin{array}{c} 0\\ 0\\ -1 \end{array}\right)+\ddot{\phi }y_{E}+\dot{\phi }{\dot{y}}_{E}\right).$$

In summary, the variational terms of the problem have now effectively been cast in the form

2.23 $$\delta \Gamma (\zeta ,\zeta^{\prime },\delta \zeta ,{\delta \zeta }^{\prime }),\;\delta \vartheta (\zeta ,\zeta^{\prime },\delta \zeta ,{\delta \zeta }^{\prime })\;\mathrm{and}\;\delta \xi (\zeta ,\zeta^{\prime },\delta \zeta ,{\delta \zeta }^{\prime }),$$

parametrized by the components of the kinematic vector ***ζ*** where

2.24 $$\begin{array}{rl} \zeta (s,t) & ={\left(\tau_{x}(s,t),\varepsilon (s,t),\tau_{z}(s,t),p{\left(s,t\right)}^{T}\right)}^{T}\\ & ={\left(\tau_{x}(s,t),\varepsilon (s,t),\tau_{z}(s,t),\theta (s,t),\psi (s,t),\phi (s,t)\right)}^{T}. \end{array}$$

### (d) Generalized coordinates

In line with d'Alembert's principle, one may state that the dynamic evolution of the system will proceed such that the virtual work contributions of equations (2.9), (2.10), (2.12), (2.16) and (2.17) will sum to zero for all admissible variations; thus

2.25 $$\delta W_{K}+\delta W_{C}+\delta W_{F}+\delta W_{M}+\delta W_{T}=0.$$

It has been shown that all of these virtual work contributions may be expressed in terms of the kinematic vector ***ζ***. In order to construct the temporal ordinary differential equations capturing the system dynamics, the components of ***ζ*** are written in a typical *s*, *t* separable form as a summation of shape functions

2.26 $$\zeta_{j}(s,t)=\sum_{k}B_{jk}(s)\;q_{jk}(t)+\zeta_{j0}(s):\;e.g.\;\theta (s,t)=\sum_{k}B_{4k}(s)q_{4k}(t)+\theta_{0}(s),$$

where ***B*** is the set of shape functions approximating the deflected state of the flexible beam continuum and *ζ*_*j*0_(*s*) denotes some optional reference distribution for each kinematic parameter from which the shape functions deform the system. *q*_*jk*_ are the time-dependent generalized coordinates that will form the state vector of this beam formulation. Note that the tensor notation is adopted throughout the remainder of this paper wherein one is to sum over all values of any index that appears *only* in multiplicative pairs. Hence, equation (2.26) is written equivalently as

2.27 $$\zeta_{j}(s,t)=B_{jk}(s)q_{jk}(t)+\zeta_{j0}(s).$$

### (e) Equations of motion

The partial derivatives of (2.25) are now taken with respect to each of the generalized coordinates *q*_*jk*_:

2.28 $$\frac{\partial W_{K}}{\partial q_{jk}}+\frac{\partial W_{C}}{\partial q_{jk}}+\frac{\partial W_{F}}{\partial q_{jk}}+\frac{\partial W_{M}}{\partial q_{jk}}+\frac{\partial W_{T}}{\partial q_{jk}}=0.$$

These terms are broken down into their constituent parts via chain differentiation in appendix Aa.

Writing the above equation (2.28) as a matrix system yields

2.29 $$\frac{\partial W_{K}}{\partial q}+\frac{\partial W_{C}}{\partial q}+\frac{\partial W_{F}}{\partial q}+\frac{\partial W_{M}}{\partial q}+\frac{\partial W_{T}}{\partial q}=0,$$

where the differential matrix operator

2.30 $$\frac{\partial }{\partial q}={\left(\frac{\partial }{\partial q_{11}},\ldots ,\frac{\partial }{\partial q_{6k}}\right)}^{T}.$$

To facilitate solution of this system, the acceleration terms in the above formulation are rearranged, factoring out any expressions that include the second-order state derivative $\ddot{q}$. This rearrangement concerns the terms ${\ddot{\vartheta }}_{\left[G\right]}$ and ${\ddot{\Gamma }}_{m[G]}$ and is detailed in appendix Af. Consequently, the kinetic virtual work **∂*W*_*T*_/∂*q*** may be split into the following parts (example matrix dimensions are also provided where *Q* is equal to the number of states in ***q***).

2.31 ∂WT∂qQ×1=−[∫0Lm(s)(∂rm[G]∂q)TQ×3(∂rm[G]∂q)3×Q+(∂ϑ[G]∂q)TQ×3e[G]3×3 Iϑ[I]3×3 e[G]T3×3 (∂ϑ[G]∂q)3×Q ds]q¨Q×1−[∫0Lm(s)(∂rm[G]∂q)TQ×3r¨m[G]∗∫3×1+(∂ϑ[G]∂q)TQ×3e˙[G]3×3 Iϑ[I]3×3 e[G]T3×3 ϑ˙[G]3×1+(∂ϑ[G]∂q)TQ×3e[G]3×3 Iϑ[I]3×3 e[G]T3×3 ϑ¨[G]∗3×1 ds]=−Mq¨−w,

where

2.32 $$\begin{array}{rl} {\ddot{\vartheta }}_{\left[G\right]}^{*} & ={\dot{\bar{\Omega }}}_{A[G]}+{\bar{\Omega }}_{A[G]}\times {\dot{\vartheta }}_{\left[G\right]}+[R_{G,A}]R_{E}\left(\dot{\theta }\dot{\psi }\left(\begin{array}{c} -s_{n}\psi \\ -\;c_{s}s_{n}\psi \\ 0 \end{array}\right)+\dot{\phi }{\dot{y}}_{E}\right), \end{array}$$

2.33 $$\begin{array}{rl} {\ddot{r}}_{m[G]}^{*} & ={\ddot{\bar{r}}}_{A[G]}+{\ddot{e}}_{\left[G\right]}^{*}\left(\begin{array}{c} a_{m}\\ 0\\ c_{m} \end{array}\right)+\int_{0}^{s}{\ddot{e}}_{\left[G\right]}^{*}\left(\begin{array}{c} \tau_{x}\\ 1+\varepsilon \\ \tau_{z} \end{array}\right)+2{\dot{e}}_{\left[G\right]}\left(\begin{array}{c} {\dot{\tau }}_{x}\\ \dot{\varepsilon }\\ {\dot{\tau }}_{z} \end{array}\right)\;d\tilde{s} \end{array}$$

2.34 $$\begin{array}{rl} \;\text{and}\;{\ddot{e}}_{\left[G\right]}^{*} & ={\ddot{\vartheta }}_{\left[G\right]}^{*}\times e_{\left[G\right]}+{\dot{\vartheta }}_{\left[G\right]}\times {\dot{\vartheta }}_{\left[G\right]}\times e_{\left[G\right]}. \end{array}$$

Here, ${\bar{\Omega }}_{A[G]}$ is the angular velocity of the coordinate system **A**.

Given this rearrangement of the dynamic terms, the final equations of motion for the nonlinear beam take the form of the ODE system:

2.35 Mq¨=−w+∂WK∂q+∂WC∂q+∂WF∂q+∂WM∂q.

Here ***M*** and ***w*** are given by equation (2.31); the remaining terms ***δW*_*K*_/*δq***, ***δW*_*C*_/*δq***, ***δW*_*F*_/*δq*** and ***δW*_*M*_/*δq*** are as defined by equations (A 1), (A 2), (A 3) and (A 4), respectively, with constituent relations feeding into these terms as detailed in appendix A(b–e).

## 3. Shape function basis

In order to implement the formulation detailed in the preceding section, one must choose a suitable set (or sets) of shape functions from which approximations of the components of ***ζ*** can be constructed. The choice of such a set is quite general, requiring only satisfaction of the essential kinematic boundary conditions of the problem. In the context of this formulation, these take the form of prescribed boundary values applied to a subset of ***ζ*** components at one or more spanwise locations along the beam. Considering equation (2.27), one may write this condition

3.1 $$\begin{array}{rl} \zeta_{j}(\bar{S},t) & =B_{jk}(\bar{S})q_{jk}(t)+\zeta_{j0}(\bar{S})=\bar{\zeta_{j}}\;\bar{S}\in [0,L]\\ & \Rightarrow \;B_{jk}(\bar{S})=0,\;\zeta_{j0}(\bar{S})=\bar{\zeta_{j}} \end{array}$$

where the value $\bar{\zeta_{j}}$ is constant. This condition holds for any constrained component of ***ζ***. It is noted here that the reference system **A**, in which this condition is cast, is a non-inertial system and thus (3.1) still admits arbitrary translation and rotation of the beam in the global coordinate system G (see, for example, the spinning beam test case of §4c).

**Figure 4.**
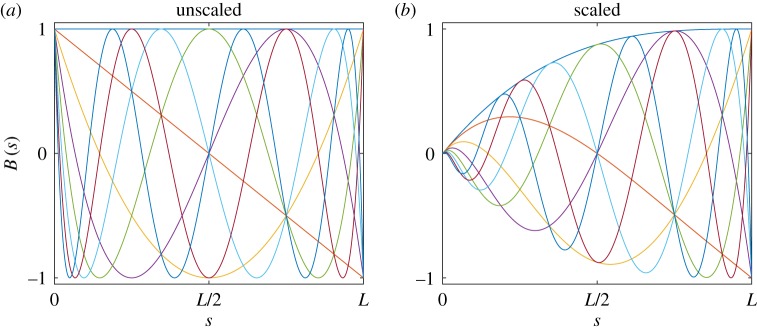
The first eight functions of the candidate shape set based on Chebyshev polynomials of the first kind. Panel (*a*) depicts the pre-multiplied set; (*b*) depicts the scaled set following application of the envelope function *E*(*s*).

A suitable set of shape functions satisfying (3.1) is depicted in figure 4*a*; these functions are based on shifted and reversed Chebyshev polynomials of the first kind. In panel (*b*), the same set is depicted following multiplication by a scaling function *E*(*s*) to bring the left side to zero (similar such scaling may be applied to the right-hand side for tip boundary conditions). This scaling procedure is applied throughout the examples of this study to constrain the applicable components of ***ζ***. A recursive generating relation for this set of functions *y*_*n*_(*x*) is given by

3.2 $$\begin{array}{rlr} B_{n}(x) & =E(s)C_{n}(x(s)),\\ x(s) & =-2\left(\frac{s}{L}\right)+1 & s\in [0,L],\\ E(s) & ={\left(\frac{s}{L}\right)}^{3}-3{\left(\frac{s}{L}\right)}^{2}+3\left(\frac{s}{L}\right),\\ C_{1}(x) & =1,\\ C_{2}(x) & =x\\ \;\mathrm{and}\;C_{n}(x) & =2xC_{n-1}(x)-C_{n-2}(x) & n\in \{1,2,\ldots ,N\}. \end{array}\}$$

Note that the use of the scaling function *E*(*s*) retains orthogonality of the underlying Chebyshev set; however, this is not a required property, nor must each component of ***ζ*** draw from the same shape function basis. Furthermore, because of the use of this scaling function, it becomes trivial to satisfy the boundary condition (3.1) for other arbitrary shape sets. However, the relative merits of different candidate shape bases are not pursued further in this study; rather the intention is to show that for the general set (3.2) satisfying the minimal essential boundary condition of the problem (3.1), the various test cases detailed in §4 can be efficiently treated (note also that throughout the cases of this paper, *ζ*_*j*0_(*s*) = 0). For similar reasons, the properties of orthogonality and natural load-dependent constraints are not discussed further here.

**Figure 5.**
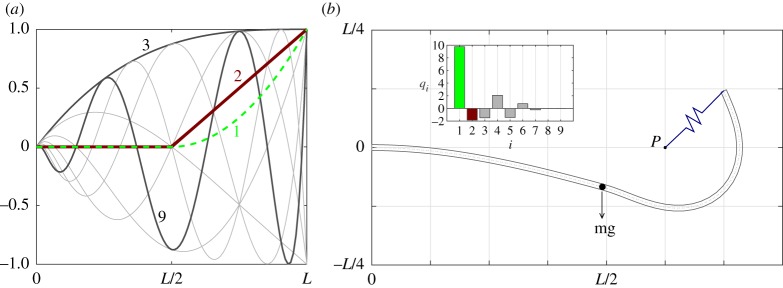
An example non-smooth beam problem. Panel (*a*) gives the modified shape function set used to embed the stiffness and mass discontinuities in the numerical problem and (*b*) depicts the predicted static deflection obtained as a consequence of the mass distribution and tip spring.

Before proceeding, it is noted that no condition on the smoothness or continuity of these shape functions has thus far been imposed; indeed any discontinuities of these sets may be treated via appropriate placement of the integration bounds of the weak formulation (2.35). Thus, discontinuous features of the physical problem may be treated by embedding the same discontinuities in the approximating shape basis. To illustrate this, consider the modelling of a straight horizontal cantilever beam with the following characteristics: firstly, the inner half running between the root and midpoint is assigned a much greater stiffness than the outer half from midpoint to tip; secondly, a point mass is fixed to the midpoint of the beam at the boundary of these two sections. One (basic) way of embedding these discontinuities into the underlying shape function set is to furnish the existing smooth functions with two additional non-smooth functions with appropriate mid-span *C*^1^ and *C*^2^ discontinuities; an example Chebyshev derived set, extended in this fashion, is depicted in figure 5*a* with the additional *C*^1^ and *C*^2^ discontinuous functions given by the dashed line and thicker line labelled 1 and 2, respectively (the remaining Chebyshev functions are indicated in increasing order by the grey curves labelled 3–9). In panel (*b*), the treatment of an example static test is shown; here a spring is connected between the beam tip and point ‘P’. The combined effects of self-weight and tip force induced by the spring cause the beam to assume the indicated shape with a clearly visible larger deformation of the more flexible outer section. The contributions of each of the shape functions to this static solution are given by the inset bar diagram in panel (*b*); the index of each bar refers to the corresponding shape function labelled in panel (*a*). Note that a more sophisticated tailoring of the underlying shape set may be employed in the efficient treatment these or other such localized characteristics; however, such a discussion is not pursued further in this study.

It is pointed out here that the treatment of such discrete features also extends to geometric discontinuities in the structure such as for a kinked beam or framed structure (see examples in [3,10,30–32]). In such cases, the discontinuity must also be represented in the kinematic description of the system. There are multiple entry points in the above formulation where this discontinuous information may be embedded. For example, one possibility is to define a generic mapping ***T***_*D*_ from the underlying Euler-referenced triad system **e**_[*E*]_ to the structure-aligned intrinsic triad system **e**_[*A*]_ used in calculating the global reference line ***Γ***_[*A*]_, i.e.

$$e_{\left[A\right]}=R_{E}(x_{E}|y_{E}|z_{E})R_{E}^{T}T_{D}.$$

Another possibility is to directly incorporate appropriate Dirac-delta functions into the initial curvature distribution ***ξ***_0_ to build up the discontinuous global geometry. These potential extensions of the formulation and their relative merits in the treatment of frame structures and other discontinuous geometries lies outside of the scope of this study.

Finally, it is worth mentioning that, alternatively, for the above examples one may of course treat smooth sub-domains of the problem breaking the discontinuities across designated element boundaries. Combined with a suitable choice of finite-element type basis set one approaches the more typical treatment of other element-based geometrically exact methods. However, in doing so one invariably moves away from the minimal state representation sought in this study; furthermore one notes that although smooth problems are exclusively treated in the examples of this study, the arbitrary smooth variation of physical parameters as displayed in the example of §4e is successfully treated in a geometrically exact manner without subdivision of the problem; this feature is novel to this nonlinear beam shapes formulation.

### 

#### (i) A note on numerical integration

Consider the numerical integration of the nonlinear beam shapes formulation. Recall the previously derived equation of motion

3.3 $$\begin{array}{rl} M\ddot{q} & =-w+\frac{\partial W_{K}}{\partial q}+\frac{\partial W_{C}}{\partial q}+\frac{\partial W_{F}}{\partial q}+\frac{\partial W_{M}}{\partial q}\\ & \;\mathrm{or}\;M(q,B(s))\;\ddot{q}=f(q,\dot{q},B(s)). \end{array}$$

Since the choice of shape functions ***B*(*s*)** is arbitrary, the constituent integral terms of ***M*** and ***f*** cannot typically be treated analytically. Specifically, the terms ***Γ***, $\dot{\Gamma }$, **∂*Γ*/∂*q***, ***M***, ***w***, **∂*W*_*K*_/∂*q***, **∂*W*_*C*_/∂*q***, **∂*W*_*F*_/∂*q*** and **∂*W*_*M*_/∂*q*** are integrated numerically over *s*∈[*O*, *L*] when evaluating the equation of motion at a particular time step. Here, numerical evaluation of these terms is accomplished using quadratic interpolation. Noting that the full system is stiff, one may compute a dynamic trajectory in time using any stiff first- or second-order solver capable of treating systems of the form (3.3). Since this method is implemented in Matlab, the in-built variable-step, variable-order stiff ODE solver ‘ODE15s’ [33] is applied to the first-order form of (3.3).

## 4. Numerical test cases

A variety of test cases, comprising benchmark tests from the literature as well as some further examples, is now presented. Each case targets particular characteristics of the geometrically nonlinear flexible beam problem. In addition to published results, the responses generated by MSC Nastran and the intrinsic beam formulation of Hodges [13,23] are in places used to validate the nonlinear beam shapes approach proposed in this paper. For all the cases, the Chebyshev shapes detailed in §3 are used in approximating *τ*_*x*_(*s*), *ε*(*s*), *τ*_*z*_(*s*), *θ*(*s*), *ψ*(*s*) and *ϕ*(*s*).

### (a) Large static deformation

**Figure 6.**
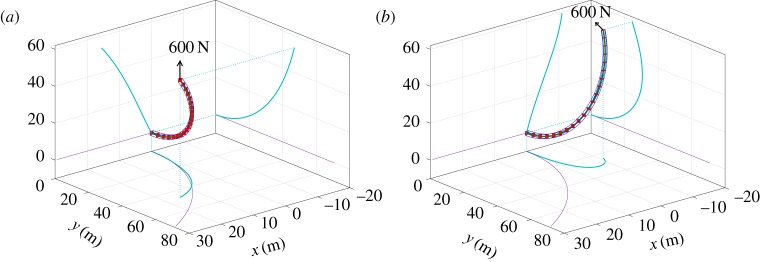
Beam deformation given (*a*) a 600 N vertical tip load and (*b*) a 600 N follower tip load.

The first test case considered is well documented in the literature and treats the large deformation of a pre-curved beam. The initial shape of the beam forms one-eighth of a circle of radius 100 m in the horizontal (*x*,*y*) plane (see the thin curves in figure 6). The beam has a 1 m^2^ cross section, a Young's modulus of *E* = 10^7^ N m^−2^ and a Poisson's ratio of zero (thus *EI*_*xx*_ = *EI*_*zz*_ = 25/3 Nmˆ2, *GJ* = 7.02885 × 10^5^ Nm rad^−1^); gravitational acceleration is not considered. The system is solved statically using the Euler mapping

4.1 $$R_{E}=\left(\begin{array}{ccc} 0 & 0 & -1\\ 0 & 1 & 0\\ 1 & 0 & 0 \end{array}\right),$$

moving the singular points away from vertical (all other test cases in this study are treated with ***R***_E_ equal to the 3 × 3 identity matrix). Two load cases are shown in figure 6, each involving the application of a 600 N tip load. In panel (*a*), the tip load is applied in the vertical *z*-direction; in panel (*b*) the same load is applied as a follower force (i.e. remaining parallel to ***e***_*z*_(*L*)). In table 1, the static tip deflection in the global coordinate system is stated as predicted by equivalent tests from a number of published sources. Results using Nastran and the intrinsic beam formulation are also displayed and a good agreement is observed.

**Table 1.** Comparison of predicted tip positions for the curved beam static test case.

source	tip position (m) *F*_vertical_	tip position (m) *F*_follower_
Bathe & Bolourchi [34]	(15.9, 47.2, 53.4)	—
Simo & Vu-Quoc [3]	(15.79, 47.23, 53.37)	—
Géradin & Cardona [35]	(15.55, 47.04, 53.50)	—
Li & Vu-Quoc [36]	(15.54, 46.85, 53.64)	—
Crisfield [30]	(15.61, 46.84, 53.71)	—
MSC Nastran	(15.56, 46.89, 53.60)	(−10.92, 24.53, 59.42)
intrinsic beam	(15.56, 46.90, 53.60)	(−10.93, 24.55, 59.41)
nonlinear beam shapes	(15.55, 46.90, 53.60)	(−10.95, 24.54, 59.41)

#### (i) Problem size

To assess the number of states required for the various methods, consider the following convergence criteria on the required number of states n*_q_:

4.2 $$n_{q}^{*}\leq \forall \;n_{q}:\{n_{q}\in N||\Gamma_{\mathrm{tip}}(n_{q})-\Gamma_{\mathrm{tip}}(2n_{q})|<0.001\},$$

i.e. doubling the number of states alters the predicted tip deflection by less than 1 mm. For the nonlinear beam shapes method, this doubling is applied to the discretization for each ***ζ*** component; for the special case of zero component shapes, one shape is added rather than doubling the set size.

Using equation (4.2), the total number of states required by the nonlinear beam shapes method, the intrinsic beam formulation and MSC Nastran is shown in table 2; results are given for both the tip vertical (figure 6*a*) and tip follower (figure 6*b*) load cases. For the current formulation, the number of shape functions assigned to *τ*_*x*_(*s*), *ϵ*(*s*), *τ*_*z*_(*s*), *θ*(*s*), *ψ*(*s*) and *ϕ*(*s*) is indicated in brackets, respectively. In addition, table 2 also provides a rough comparison of the required computational effort in treating these systems. To achieve this, the follower and vertical load cases are run as dynamic simulations over a period of 60 s. A large stiffness proportional damping is used (***F***_*K*_ =  − ***K*Δ*ξ***, $F_{C}=-K\Delta \dot{\xi }$) such that the responses are settled to their respective equilibria by the end of the 60 s simulation. The nonlinear beam shapes and intrinsic beam methods are run in Matlab using the stiff ODE solver ‘ODE15s’ [33]; no Jacobian information is fed to the solver for the former method. Nastran is run using its in-built SOL400 solution routine with adaptive time stepping. Owing to the large deformations involved in this test case, it proved difficult to obtain an accurate Nastran transient response for the follower 600 N tip load. Hence only the Nastran benchmark time based upon the vertical load case is provided in table 2. Because of the differences in the numerical integration schemes only a crude comparison of computational speed can be inferred here. Looking to the results of table 2, the nonlinear beam shapes formulation required an order of magnitude fewer states, accompanied by a significantly reduced simulation time when compared with the other methods. Of particular note is that no shear or extensional states were required by the nonlinear beam shapes formulation in meeting the convergence criterion in this example. Indeed, this is the case for a majority of the example problems of this study wherein capturing large nonlinear deformations results in the treatment of flexible and slender structures where shear effects are not significant. One advantage of this formulation is that the shear and extensional compliances can easily be removed by simply not assigning shapes to *τ*_*x*_(*s*), *ϵ*(*s*) and *τ*_*z*_(*s*). Note however that the shallow arch example of the following section introduces a test case for which these additional shear/extensional flexibilities do prove influential on the resulting structural response.

**Table 2.** Static degrees of freedom required in satisfying the convergence criterion (4.2), and computational benchmarks.

method and load case	problem discretization	static doFs	dynamic solution time (s)
Nastran (vertical)	22 elements	132	40.1
Nastran (follower)	60 elements	360	—
intrinsic beam (vertical)	31 elements	186	132
intrinsic beam (follower)	69 elements	414	760
nonlinear beam shapes (vertical)	11 shapes (0, 0, 0, 4, 4, 3)	11	3.26
nonlinear beam shapes (follower)	15 shapes (0, 0, 0, 5, 5, 5)	15	4.25

### (b) Shallow arch example

This test case focuses on the response of a pin-jointed shallow arch subject to a distributed radial load. The motivating features of this test case are two-fold. Firstly, the treatment of non-cantilever boundary conditions provides a further example of the construction of equivalent kinematic boundary conditions in the context of this formulation. Secondly, the loaded arch provides a system for which the shear/extension states prove influential on the observed response.

The shallow arch is based upon the pre-curved beam of the previous example. The cross-sectional properties remain the same; however, the radius of curvature is now reduced to 20 m—consequently reducing the beam length over the 45° arc. This curved beam is supported on two pin joints to form an un-stressed arch to which a radial load is applied. This problem is planar and oriented in the (*y*,*z*)–plane; thus *ψ*(*s*) = *ϕ*(*s*) = *τ*_*x*_(*s*) = 0, a condition achieved simply by assigning no shape functions to the 1st, 5th and 6th components of ***ζ***. The remaining *θ*(*s*), *ε*(*s*) and *τ*_*z*_(*s*) kinematic parameters are unconstrained at both boundaries of the arch, thus the unscaled shape set of figure 4*a* is selected for these components. ***Γ***_[*G*]_(0) = ***Γ***_0_ and ***Γ***_[*G*]_(*L*) = ***Γ***_*L*_ correspond to the displacement constraints at each end of the arch. ***Γ***_[*G*]_(0) = ***Γ***_0_ is implicitly satisfied by equation (2.1) where ${\bar{r}}_{A[G]}=\Gamma_{0}$; to constrain the right-hand boundary equation (2.28) is furnished with the additional energy term

4.3 $$\delta W_{\Upsilon }=\Upsilon (\Gamma_{\left[G\right]}(L)-\Gamma_{L})\delta \Gamma_{\left[G\right]}(L),$$

where the multiplier *Υ* is given a sufficiently large value to constrain ***Γ***_[*G*]_(*L*).

Application of a distributed radial load will cause this arch to buckle. Despite the simplicity of the configuration, there are a number of distinct buckling behaviours that may be observed for this system. In [37], Pi *et al*. classify these as snap-through and bifurcation buckling modes, the latter of which is typically asymmetric in nature. The specific buckling characteristic observed is related to the non-dimensional ‘shallowness’ parameter *λ*. For a rigid-pinned circular arch such as that treated in this example this parameter takes the form

4.4 $$\lambda =\frac{L^{2}\sqrt{A}}{4R\sqrt{I_{1}}},$$

where *A* is the cross-sectional area of the beam, *I*_1_ is the second moment of area and *R* is the radius of curvature. This yields a lambda value of 10.68 for this test case.

**Figure 7.**
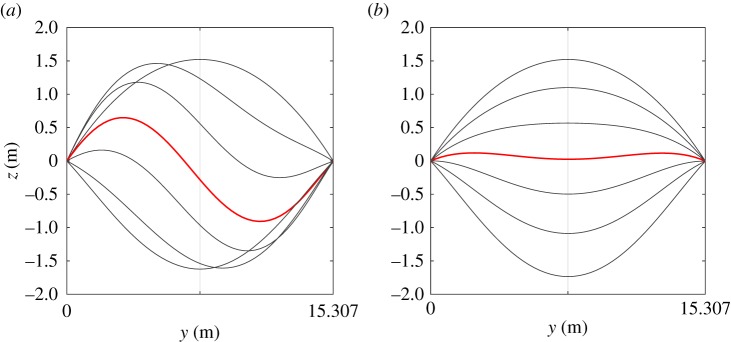
Arch buckling following application of a distributed load. Panel (*a*) excludes shear and extensional states; (*b*) includes shear/extension. In both cases, five shapes are used for each active component of ***ζ***(*s*).

Looking now to figure 7, the shape of the shallow arch is shown following application of a radial load, and snapshots of the deformation of the arch as it buckles are depicted. In panel (*a*), a distributed load of 7500 N m^−1^ is applied; no shear or extensional compliance is permitted and the arch is observed to buckle under an asymmetric bifurcation. This agrees with analytic results for a rigidly supported arch with *λ* above the critical value of approximately 9.2. The effect of elastically compliant supports are also considered in [37]. It is shown that as the flexibility of these supports is increased, the effective *λ* is reduced, allowing for symmetric snap-through behaviour. Rather than directly modifying the support flexibility in this example, the addition of shear and extensional states is used to produce an alternative source of radial and axial compliance to the system. In figure 7*b*, five shapes are assigned to each of the extensional *ε*(*s*) and shear *τ*_*z*_(*s*) parameters; the applied load is reduced to 5500 N m^−1^ in light of the increased flexibility of the arch. Looking at the snapshots of the arch deformation, one observes that the addition of shear and extensional states is indeed sufficient to capture the existence of a symmetric snap-though mode.

### (c) Rotating pre-curved beam

The following two examples are based upon the test beam treated in Pai [31]. The parameters are summarized in table 3. For the first test case, the beam is oriented in the global *y*-direction (***e***_*y*[*A*]_(0) = (0, 1, 0)^T^) and assigned a constant pre curvature *κ*_*x*_ =  − (3*π*)/(18*L*) m^−1^.

**Table 3.** Pai beam parameters from [31].

parameter	value	units
length	0.479	m
width	5.08 × 10^−2^	m
height	4.5 × 10^−4^	m
*EI*_*xx*_	4.899184 × 10^−2^	Nm^2^
*EI*_*zz*_	6.243471	Nm^2^
*GJ*	7.164459 × 10^−2^	Nm rad^−1^
mass per length	1.012698 × 10^−1^	kg m^−1^
torsional inertia per length	2.178012 × 10^−1^	kg m

**Figure 8.**
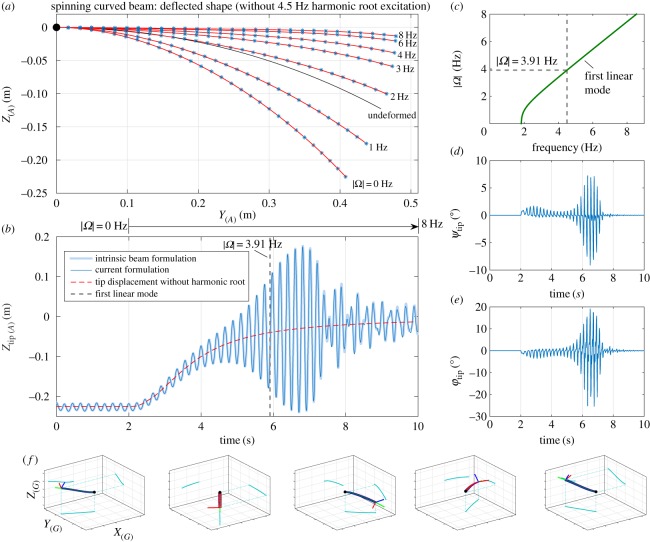
Predicted responses for the pre-curved spinning beam test case.

This pre-curved geometry is shown in figure 8*a* by the curve labelled ‘undeformed’. The additional results are obtained by first allowing the beam to deflect under its own weight (0 Hz curve). Then the root is spun in the global *z*-direction at a number of angular velocities. The settled deformation as predicted by the shape function formulation is depicted by the labelled red lines for rotation speeds of 1, 2, 3, 4, 6 and 8 Hz. Corresponding data points from [31] are overlaid onto these curves illustrating a very close agreement. For the second phase of this test, the root of the beam is fed a 4.5 Hz harmonic excitation in the vertical direction of amplitude ± 2 cm in addition to the applied angular rotation. To help with visualization, the smaller plots at the bottom of figure 8 (panel (*f*)) illustrate the oscillating and spinning motion of this system in the global coordinate system (*X*_[*G*]_, *Y*_[*G*]_, *Z*_[*G*]_). A stiffness proportional, viscous damping law is assumed at 1% of the structural stiffness; i.e. $F_{C}(s,\Delta \xi ,\dot{\xi })=-0.01K(s)\dot{\xi }(s)$ in equation (2.10). The blue solid line in panel (*b*) shows the tip response for 10 s of this test case. At first, the rotational velocity of the system is zero and thus the initial beam response is due purely to the root vertical oscillation. The settled response to this excitation is shown for the first 2 s of the time series. Then for 2 ≤ *t* ≤ 10 the rotational velocity |***Ω***_[*G*]_| is ramped linearly from 0 to 8 Hz. Over this time, the beam tip continues to oscillate and remains centred about the dashed red line which corresponds to the static solution without the root vertical oscillation (compare with panel (*a*)). At the onset of the rotational sweep (*t* = 2) one also notes both an out-of-plane and torsional component of the beam response (depicted in panels (*d*) and (*e*) by the non-zero *ψ*(*L*) and *ϕ*(*L*) components of the response). This illustrates the expected gyroscopic effects resulting from large local rotational motion of the structure in the rotating beam reference frame.

Looking at these responses, one notes that the amplitude of oscillation grows as the root angular velocity is increased reaching a maximum before dropping off again. Physically, one is observing here the increasing centrifugal dynamic component having a stiffening effect on the flexible system and consequently increasing the frequencies at which the flexible modes reside (see, for example, the modelling studies of [38–40] for treatments of the centrifugal stiffening effect in the context of simple rotating systems). This stiffening is illustrated in panel (*c*) where the relationship between the root angular velocity and the first modal linear frequency is depicted. For a rotational velocity of 3.91 Hz—occurring at *t* = 5.91 s in the simulated test case and indicated by the vertical line in panel (*b*)—this modal frequency is equal to 4.5 Hz and therefore equal to the frequency of root excitation of the system. Given a small amplitude excitation and sufficiently slow sweep, the maximum response amplitude would be observed close to this angular velocity. However, for the level of harmonic excitation applied in this example the amplitude of the system response is sufficient to push into the nonlinear regime of the system dynamics and consequently the resonant frequency at the observed amplitudes will not coincide with this linear frequency; coupled with the relatively quick traversal through this resonant region one observes a delayed maximal response at a larger rotational frequency. To verify this case, the same test was performed using the intrinsic beam model; the resulting tip deflection is also depicted in the background of panel (*b*) by the thicker background line. Here, one sees that the obtained envelope of oscillation is almost identical between the two methods and that the time series match well, deviating only very slightly towards the end of the simulation.

### (d) Harmonically excited vertical cantilever

**Figure 9.**
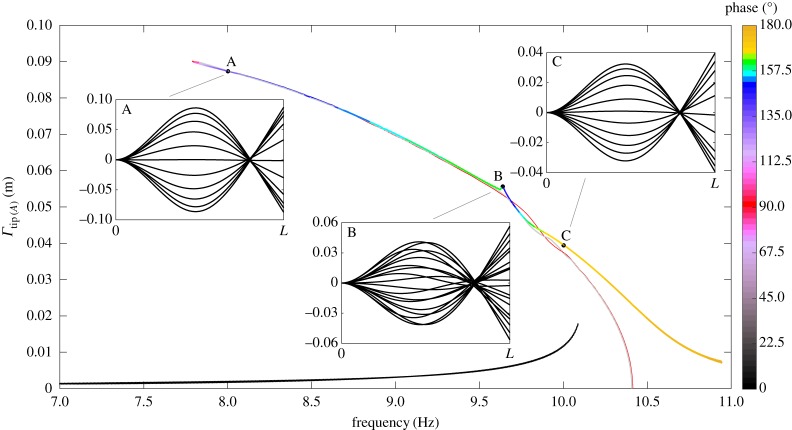
Example frequency response diagram approximated via numerical simulation of the system for a 0.005% stiffness proportional damping and ±1 mm base excitation. Estimates of the underlying nonlinear normal mode, as predicted by the current formulation and intrinsic beam model, are indicated by the thin red and thin grey curves, respectively. Snapshots of the beam oscillations are provided at points A (8 Hz), B (9.64 Hz) and C (10 Hz) along the upper periodic branch.

This next test case also concerns the beam detailed in the previous example. A harmonic root excitation is again fed to this system, this time in isolation. The beam configuration matches the corresponding experimental/numerical study conducted in [31]. Specifically, the beam is oriented pointing vertically upwards and subject to its own weight. The transverse harmonic oscillation applied to the beam root acts in the ± ***e***_*z*_(0) direction and has an amplitude of ±1 mm. Structural damping is again stiffness proportional and set to a very light value of 0.005% of the beam stiffness. Two slow frequency sweeps of the root harmonic excitation are performed over the range 7–11 Hz in both the forward and reverse directions. The amplitude of tip oscillation relative to the base position, as this excitation frequency is varied, is plotted by the thicker curves in figure 9. These curves form approximations of the upper and lower stable periodic branches of the frequency response function for this level of root excitation and damping (the curves are clipped off at points where a loss of stability is observed). The colour at any location along these curves corresponds to the observed phase shift between the tip response and base excitation as indicated by the bar to the right of the figure.

The phase of the periodic branch formed by the two stable parts and unstable part (not indicated) traverses a large portion of the 0–180° phase envelope as the excitation frequency is increased over the range illustrated. Three sub-plots are also provided, illustrating snapshots of the beam deflection at three sample locations along the upper branch labelled A–C. In the 8 Hz and 10 Hz sub-plots, the oscillatory response of the flexible beam is observed to correlate closely with the second linear mode shape of the system with a clearly defined nodal point observable. At the 9.64 Hz sample location, this vibration mode and characteristic nodal point is not cleanly observed. Looking to the upper branch topology at this sample location, one notes a localized ‘kink’ in the periodic curve and accompanying variation in phase lag. This feature corresponds to a 3 : 1 resonance between the second and third nonlinear flexible modes (for reference, the first three linear modal frequencies for the vertically oriented beam are 1.44, 10.42 and 29.55 Hz). Note that the precise topology of the periodic structure around this resonance point is not known, as the illustrated curve simply reflects the transient response as the base excitation sweep traverses this region; however, clearly the current formulation is capable of capturing these higher-order resonances. For further discussion of such resonant branch phenomena see [41] wherein the out-of-plane resonance of a similar square cross-sectioned flexible beam is detailed.

Following the upper periodic curve towards the left, one notes the phase lag approaches (and passes through) 90°. Nonlinear vibration theory dictates that (providing the strain rate damping law is linear) this point of quadrature at 90° coincides with a point on the nonlinear normal mode (or backbone curve) of the flexible system. By allowing the unforced system to decay from this point of quadrature, one may trace a portion of this backbone curve by sampling the transient frequency and amplitude of the decaying response. The resulting backbone section is shown in the figure by the thinner red curve, beginning at the quadrature point and decaying in amplitude until the linear frequency at 10.42 Hz is met (note that the exact backbone curve for this system extends indefinitely beyond this quadrature point). The overall curve has a clear left tilt in the figure and thus indicates a softening of the second nonlinear normal mode as the amplitude of oscillation is increased (also observed in [31]). One notes that the resonant ‘kink’ previously observed in the upper branch also helps shape the transient decay and subsequent backbone prediction in the region 9.5–10 Hz.

As in the previous examples validation is sought via comparison with the intrinsic beam formulation; performing an identical decay from the same initial state yields a second backbone approximation predicted by the latter method and indicated by the grey curve. One notes close agreement between each backbone result, both tracing a similar shape and indicating the nonlinear softening effect. Note that there is some slight difference between the two results in the 9.5–10 Hz range; however, due to the speed of decay it is unlikely that either method closely captures the localized backbone structure in this region. For the precise tracking of this backbone curve, one could employ more sophisticated methods based upon shooting or pseudo-arclength continuation [42–44], however, this is not pursued here.

### (e) Wind turbine blade

**Figure 10.**
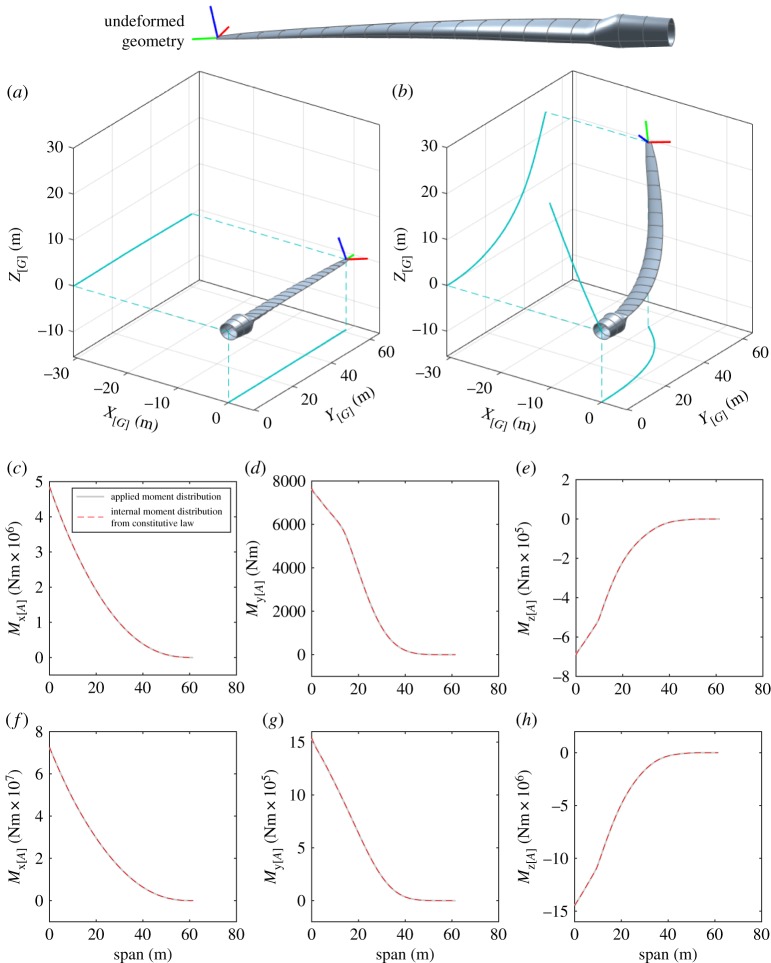
Example deflections for the wind turbine test case.

The final test case details the static deformation of a wind turbine blade based upon the ‘NREL offshore 5-MW baseline wind turbine’ detailed in the technical report of Jonkman *et al.* [45] with supplementary parameters taken from the pre-design study of Kooijman *et al.* [46] (part of the Dutch Offshore Wind Energy Converter (DOWEC) project). The blade has a span of 61.5 m from its hub attachment point to the tip; the unloaded structural geometry exhibits variable material properties, pre curvature and twist from its root to tip. For the specific turbine blade of this test, arbitrary parametrized functions are fitted to the data and used to concisely express these span-dependent quantities; these functions are detailed in table 4. A depiction of the undeformed blade geometry is provided at the top of figure 10. For this test, the turbine blade is oriented such that

4.5 $$[R_{G,A}]=\left(\begin{array}{ccc} \cos(20) & 0 & -\sin(20)\\ 0 & 1 & 0\\ \sin(20) & 0 & \cos(20) \end{array}\right).$$

***R***_E_ is equal to the identity matrix. A distributed load is applied to this structure along the reference line ***Γ***_*G*_(*s*). The load has an elliptical profile and acts normal to the blade chord line, i.e.

4.6 $$F_{G}(s)=\bar{f}\sqrt{\left(1-{\left(\frac{s}{L}\right)}^{2}\right)}e_{z}(s).$$

Two load cases corresponding to values of $\bar{f}=(2.4\times 10^{5}/L)\;{\mathrm{Nm}}^{-1}$ and $\bar{f}=(4\times 10^{6}/L)\;{\mathrm{Nm}}^{-1}$ in expression (4.6) are applied to the blade in panels (*a*) and (*b*) of figure 10, respectively. Each panel shows the static deformation achieved via dynamic simulation of the system, allowing sufficient time for transient motion to settle; a conservative choice of eight Chebyshev polynomials per Euler angle was used for the treatment of this test. For the first load case of panel (*a*), the applied load is just sufficient to remove the pre-curvature from the turbine blade. For the second load case of panel (*b*), the load produces an extreme deflection of the structure far in excess of any physically realizable load; this latter case is used to demonstrate the ability to solve the geometrically exact problem for large deformations of the generic non-prismatic structure. To verify these deformations both the net applied moment (a consequence of the applied load distribution ***F***_*G*_) and the internal moment (calculated from the Euler-Bernoulli constituent relation) are compared at each spanwise location in the range *s*∈[0, *L*]. In panel (*c*), the out-of-plane (***e***_*x*_) component of the external (solid) and internal (dashed) moment distributions are plotted for the first load case; panels (*d*) and (*e*) depict the twist (***e***_*y*_) and in-plane (***e***_*z*_) components, respectively. Panels (*f*–*h*) provide the same three plots for the second, larger load case. Correct solution of the static problem may be verified by noting the close agreement indicated in these sub plots.

**Table 4.** Wind turbine blade parameters.

span-dependent function	coefficients
length: *L* = 61.5 (m)	
$\begin{array}{l} EI_{xx}=a_{1}+a_{2}x^{1/a_{3}}+a_{4}x+a_{5}x^{2}+a_{6}x^{3}+a_{7}x^{4}\\ \left(Nm^{2}\right)\;x=1-s/L \end{array}$	$\begin{array}{ll} a_{1}=12.04 & a_{2}=7.4296\\ a_{3}=5.9294 & a_{4}=0.6791\\ a_{5}=6.3115 & a_{6}=-2.6386\\ a_{7}=-0.9242 \end{array}$
$\begin{array}{l} GJ=a_{1}+a_{2}x^{1/a_{3}}+a_{4}x+a_{5}x^{2}+a_{6}x^{3}+a_{7}x^{4}\\ {\left(\mathrm{Nm}\;\mathrm{rad}\right)}^{-1}\;x=1-s/L \end{array}$	$\begin{array}{ll} a_{1}=12.15 & a_{2}=6.8864\\ a_{3}=3.5466 & a_{4}=-1.2809\\ a_{5}=2.0892 & a_{6}=2.8858\\ a_{7}=0.0322 \end{array}$
$\begin{array}{l} EI_{zz}=a_{1}+a_{2}x^{1/a_{3}}+a_{4}x+a_{5}x^{2}+a_{6}x^{3}+a_{7}x^{4}\\ \left(Nm^{2}\right)\;x=1-s/L \end{array}$	$\begin{array}{ll} a_{1}=15.43 & a_{2}=7.2698\\ a_{3}=4.0351 & a_{4}=0.1553\\ a_{5}=-0.2459 & a_{6}=0.3072\\ a_{7}=0.4667 \end{array}$
$\begin{array}{l} m=a_{1}x^{1/2}(1+a_{2}x^{2}+a_{3}x^{3}+a_{4}x^{4}+a_{5}x^{5}+a_{6}x^{6})\\ {\left(\mathrm{kg}\;m\right)}^{-1}\;x=1-s/(L+0.2) \end{array}$	$\begin{array}{ll} a_{1}=250 & a_{2}=-1.4119\\ a_{3}=17.8617 & a_{4}=-21.5880\\ a_{5}=-9.7330 & a_{6}=17.0449 \end{array}$
$\begin{array}{l} \theta_{0}=-\tan^{-1}((\sum_{n=0}^{6}a_{n}x^{n})(\tan^{-1}(s-14)/\pi +0.5))\\ \left(\mathrm{rad}\right)\;x=(s-30.75)/18.02 \end{array}$	$\begin{array}{ll} a_{0}=0.029954 & a_{1}=0.039748\\ a_{2}=-0.0031189 & a_{3}=-0.0074728\\ a_{4}=0.0080149 & a_{5}=0.00047782\\ a_{6}=-0.0021779 & \end{array}$
*ψ*_0_ = 0 (rad)	—
$\begin{array}{l} \phi_{0}=(13.5-\sum_{n=0}^{9}a_{n}x^{n})\;(\tan^{-1}(s-10)/\pi +0.5)-13.5\\ \left(\mathrm{rad}\right)\;x=(s-30.75)/18.02 \end{array}$	$\begin{array}{ll} a_{0}=6.5963 & a_{1}=-5.3307\\ a_{2}=-0.71825 & a_{3}=0.73322\\ a_{4}=2.6739 & a_{5}=-1.2031\\ a_{6}=-1.7149 & a_{7}=0.80577\\ a_{8}=0.30529 & a_{9}=-0.14539 \end{array}$

## 5. Conclusion

This paper has detailed the development and application of a nonlinear beam shapes approach capable of the low-order geometrically exact representation of a flexible beam; the formulation is based upon the shape-based discretization of attitude and incremental shear kinematic quantities distributed along the flexible structure. The development of the describing equations of motion was demonstrated for an example Euler angle parametrization and coupled to a set of Chebyshev polynomials of the first kind, modified to admit the generalized kinematic condition. The formulation was subsequently applied to a variety of test cases including the modelling of planar and non-planar transient dynamics, the prediction of static equilibria and the treatment of simple beam, pre-curved and non-prismatic structures, all within a geometrically nonlinear context. The shape functions used are generic and may be tailored to the characteristics of the modelled physical system. Complementary Nastran and intrinsic beam calculations were used to verify specific results throughout these tests; altogether the formulation of this study demonstrated the accurate treatment of the considered test cases, consistently achieved using a small set of states, representing an order of magnitude reduction in problem size over its element-based counterparts. This low-order formulation admits the efficient treatment of flexible beam analyses for which the size of system generated by traditional element-based representations would incur a significant computational penalty; such examples include structural optimization problems over large parametric design spaces, sensitivity and uncertainty quantification analyses, and numerical continuation methods exploring limiting dynamic behaviours.

## Supplementary Material

Supplementary Data (On the geometrically exact low-order modelling of a flexible beam: formulation and numerical tests)

## Note

1 Note that this curvature does not depend on the axial extension *ε*> and therefore represents the rotational gradient per undeformed arclength *s*.

## Data accessibility

Datasets supporting the depicted results of this study are provided in the accompanying electronic supplementary material.

## Appendix A

**(a) Virtual work derivatives**

**(i) Strain**

From equation (2.9),

A 1 $$\frac{\partial W_{K}}{\partial q_{jk}}=\int_{0}^{L}F_{K}{\left(s,\Delta \xi \right)}_{i}\frac{\partial \xi_{i}}{\partial q_{jk}}\;ds,\;\mathrm{where}\;\frac{\partial \xi_{i}}{\partial q_{jk}}=\frac{\partial \xi_{i}}{\partial \zeta_{j}}\frac{\partial \zeta_{j}}{\partial q_{jk}}+\frac{\partial \xi_{i}}{\partial \zeta_{j}^{\prime }}\frac{\partial \zeta_{j}^{\prime }}{\partial q_{jk}},$$

*F*_*K*_(*s*, **Δ*ξ***)_*i*_ denoting the *i*th component of ***F*_*K*_**.

**(ii) Damping**

From equation (2.10),

A 2 $$\frac{\partial W_{C}}{\partial q_{jk}}=\int_{0}^{L}F_{C}{\left(s,\Delta \xi ,\dot{\xi }\right)}_{i}\frac{\partial \xi_{i}}{\partial q_{jk}}\;ds,\;\mathrm{where}\;{\dot{\xi }}_{i}=\left(\frac{\partial \xi_{i}}{\partial \zeta_{j}}\frac{\partial \zeta_{j}}{\partial t}+\frac{\partial \xi_{i}}{\partial \zeta_{j}^{\prime }}\frac{\partial \zeta_{j}^{\prime }}{\partial t}\right).$$

**(iii) Applied force**

From equation (2.12),

A 3 $$\frac{\partial W_{F}}{\partial q_{jk}}=\int_{0}^{L}F_{\left[G\right]}(s)\cdot \frac{\partial r_{F[G]}}{\partial q_{jk}}\;ds,\;\mathrm{where}\;\frac{\partial r_{F[G]}}{\partial q_{jk}}=\frac{\partial e_{\left[G\right]}}{\partial q_{jk}}\left(\begin{array}{c} a_{F}\\ 0\\ c_{F} \end{array}\right)+\frac{\partial \Gamma_{\left[G\right]}}{\partial q_{jk}}.$$

**(iv) Applied moment**

From equation (2.16),

A 4 $$\frac{\partial W_{M}}{\partial q_{jk}}=\int_{0}^{L}M_{\left[G\right]}(s)\cdot \frac{\partial \vartheta_{\left[G\right]}}{\partial q_{jk}}\;ds.$$

**(v) Kinetic term**

From equation (2.17),

A 5 $$\begin{array}{rl} & \frac{\partial W_{T}}{\partial q_{jk}}=\int_{0}^{L}m{\ddot{r}}_{m[G]}\cdot \frac{\partial r_{m[G]}}{\partial q_{jk}}+({\dot{e}}_{\left[G\right]}I_{\vartheta [I]}e_{\left[G\right]}^{T}{\dot{\vartheta }}_{\left[G\right]}+e_{\left[G\right]}I_{\vartheta [I]}e_{\left[G\right]}^{T}{\ddot{\vartheta }}_{\left[G\right]})\cdot \frac{\partial \vartheta_{\left[G\right]}}{\partial q_{jk}}\;ds\\ & \;\mathrm{where}\;\frac{\partial r_{m[G]}}{\partial q_{jk}}=\frac{\partial e_{\left[G\right]}}{\partial q_{jk}}\left(\begin{array}{c} a_{m}\\ 0\\ c_{m} \end{array}\right)+\frac{\partial \Gamma_{\left[G\right]}}{\partial q_{jk}},\;{\ddot{r}}_{m[G]}={\ddot{e}}_{\left[G\right]}\left(\begin{array}{c} a_{m}\\ 0\\ c_{m} \end{array}\right)+{\ddot{\Gamma }}_{\left[G\right]}. \end{array}$$

To complete the terms (A 1–A 5), one requires definition of the partial derivatives ∂***Γ***_[*G*]_/∂*q*_*jk*_, ∂^2^***Γ***_[*G*]_/∂*t*^2^, ∂***𝜗***_[*G*]_/∂*q*_*jk*_, ∂***𝜗***_[*G*]_/∂*t*, ∂^2^***𝜗***_[*G*]_/∂*t*^2^, ∂**e**_[*G*]_/∂*q*_*jk*_, ∂**e**_[*G*]_/∂*t*, ∂^2^**e**/∂*t*^2^, ∂*ξ*_*i*_/∂*ζ*_*j*_, ∂*ξ*_*i*_/∂*ζ*′_*j*_, ∂*ζ*_*j*_/∂*q*_*jk*_, ∂*ζ*′_*j*_/∂*q*_*jk*_, ∂*ζ*_*j*_/∂*t*, ∂*ζ*′_*j*_/∂*t*, ∂^2^*ζ*_*j*_/∂*t*^2^. These are given in the following appendix sections A(b–e).

**(b) *ζ* Derivatives**

A 6 $$\begin{array}{rl} & \zeta_{j}(s,t)=B_{jk}(s)q_{jk}(t)+\zeta_{j0}(s)\\ \;\mathrm{and}\; & \frac{\partial \zeta_{j}}{\partial q_{jk}}=B_{jk},\;\frac{\partial \zeta_{j}^{\prime }}{\partial q_{jk}}=B_{jk}^{\prime },\;\frac{\partial \zeta_{j}}{\partial t}=B_{jk}{\dot{q}}_{jk},\;\frac{\partial \zeta_{j}^{\prime }}{\partial t}=B_{jk}^{\prime }{\dot{q}}_{jk},\;\frac{\partial {\;}^{2}\zeta_{j}}{\partial t^{2}}=B_{jk}{\ddot{q}}_{jk}. \end{array}\}$$

**(c) *ξ* Derivatives**

The partial derivatives ∂*ξ*_*i*_/∂*ζ*_*j*_ and ∂*ξ*_*i*_/∂*ζ*′_*j*_ follow simply from differentiation of

A 7 $$\xi =\left(\begin{array}{c} \tau_{x}\\ \varepsilon \\ \tau_{z}\\ \kappa_{x}\\ \kappa_{y}\\ \kappa_{z} \end{array}\right)=\left(\begin{array}{c} \tau_{x}\\ \varepsilon \\ \tau_{z}\\ R_{E}\left[\begin{array}{c} \psi^{\prime }\;c_{s}s_{n}\theta s_{n}\phi +\theta^{\prime }\;c_{s}s_{n}\phi \\ \phi^{\prime }-\psi^{\prime }s_{n}\theta \\ -\psi^{\prime }\;c_{s}s_{n}\theta \;c_{s}s_{n}\phi +\theta^{\prime }s_{n}\phi \end{array}\right] \end{array}\right)\;\mathrm{with}\;\mathrm{respect}\;\mathrm{to}\;\zeta =\left(\begin{array}{c} \tau_{x}\\ \varepsilon \\ \tau_{z}\\ \theta \\ \psi \\ \phi \end{array}\right)\;\mathrm{and}\;\zeta^{\prime }=\left(\begin{array}{c} \tau_{x}^{\prime }\\ \varepsilon^{\prime }\\ \tau_{z}^{\prime }\\ \theta^{\prime }\\ \psi^{\prime }\\ \phi^{\prime } \end{array}\right).$$

**(d) *𝜗* Derivatives**

***δ𝜗***_[*A*]_(***δζ***) is given by equation (2.21). Thus, each partial derivative of ***𝜗***_[*A*]_ follows from differentiation of ***δζ*** with respect to the variable of interest (see A(b)). ${\ddot{\vartheta }}_{\left[A\right]}(\ddot{\zeta })$

is given by equation (2.22). Similarly, $\ddot{\zeta }$ is given in A(b).

In the global coordinate system,

$${\delta \vartheta }_{\left[G\right]}={\delta \beta }_{A[G]}+[R_{G,A}]{\delta \vartheta }_{\left[A\right]},{\ddot{\vartheta }}_{\left[G\right]}={\dot{\bar{\Omega }}}_{A[G]}+[R_{G,A}]{\ddot{\vartheta }}_{\left[A\right]}+\frac{d\left[R_{G,A}\right]}{dt}{\dot{\vartheta }}_{\left[A\right]}$$

where

A 8 $$\frac{d\left[R_{G,A}\right]}{dt}={\bar{\Omega }}_{A[G]}\times [R_{G,A}].$$

***β***_*A*_ is the rotation vector of the reference frame **A** and ${\bar{\Omega }}_{A}={\dot{\beta }}_{A}$.

**(e) *Γ*** Derivatives

A 9 $$\begin{array}{rl} {\delta e}_{\left[G\right]} & ={\delta \vartheta }_{\left[G\right]}\times e_{\left[G\right]},\;{\ddot{e}}_{\left[G\right]}={\ddot{\vartheta }}_{\left[G\right]}\times e_{\left[G\right]}+{\dot{\vartheta }}_{\left[G\right]}\times {\dot{\vartheta }}_{\left[G\right]}\times e_{\left[G\right]}, \end{array}$$

A 10 $$\begin{array}{rl} \Gamma_{\left[G\right]} & ={\bar{r}}_{A[G]}+\int_{0}^{s}e_{\left[G\right]}\left(\begin{array}{c} \tau_{x}\\ 1+\varepsilon \\ \tau_{z} \end{array}\right)\;d\tilde{s}={\bar{r}}_{A[G]}+\int_{0}^{s}e_{\left[G\right]}\tau \;d\tilde{s} \end{array}$$

A 11 $$\begin{array}{rl} \;\text{and}\;{\delta \Gamma }_{\left[G\right]} & ={\dot{\bar{r}}}_{A[G]}+\int_{0}^{s}{\delta e}_{\left[G\right]}\tau +e_{\left[G\right]}\delta \tau \;d\tilde{s},\;{\ddot{\Gamma }}_{\left[G\right]}={\ddot{\bar{r}}}_{A[G]}+\int_{0}^{s}{\ddot{e}}_{\left[G\right]}\tau +2{\dot{e}}_{\left[G\right]}\dot{\tau }+e_{\left[G\right]}\ddot{\tau }\;d\tilde{s}. \end{array}$$

**(f) Rearrangement of kinetic terms**

Rearrangement of the second-order derivative terms into the form $\left(a\ddot{q}+b\right)$ is required to cast this formulation as the explicit system of ODEs (2.35). These rearrangements are detailed here.

A 12 $$\begin{array}{rl} {\delta \vartheta }_{\left[I\right]}(\delta \zeta ) & =\delta \theta \left(\begin{array}{c} \;c_{s}s_{n}\psi \\ -s_{n}\psi \\ 0 \end{array}\right)+\delta \psi \left(\begin{array}{c} 0\\ 0\\ -1 \end{array}\right)+\delta \phi y_{E},\\ {\dot{\vartheta }}_{\left[I\right]}(\zeta ,e_{\chi }) & =\frac{\partial \vartheta }{\partial \zeta_{j}}\frac{\partial \zeta_{j}}{\partial q_{jk}}\frac{\partial q_{jk}}{\partial t}=\dot{\theta }\left(\begin{array}{c} \;c_{s}s_{n}\psi \\ -s_{n}\psi \\ 0 \end{array}\right)+\dot{\psi }\left(\begin{array}{c} 0\\ 0\\ -1 \end{array}\right)+\dot{\phi }\;y_{E}\\ {\ddot{\vartheta }}_{\left[I\right]}(\zeta ,e_{\chi }) & =\frac{\partial \vartheta }{\partial \zeta_{j}}\frac{\partial \zeta_{j}}{\partial q_{jk}}\frac{\partial {\;}^{2}q_{jk}}{\partial t^{2}}+\frac{\partial }{\partial t}\left(\frac{\partial \vartheta }{\partial \zeta_{j}}\frac{\partial \zeta_{j}}{\partial q_{jk}}\right)\frac{\partial q_{jk}}{\partial t}\\ & =\frac{\partial \vartheta }{\partial \zeta_{j}}\frac{\partial \zeta_{j}}{\partial q_{jk}}\frac{\partial {\;}^{2}q_{jk}}{\partial t^{2}}+\frac{\partial }{\partial \zeta_{j}}\left(\frac{\partial \vartheta }{\partial \zeta_{j}}\frac{\partial \zeta_{j}}{\partial q_{jk}}\right)\frac{\partial \zeta_{j}}{\partial t}\frac{\partial q_{jk}}{\partial t}+\frac{\partial }{\partial e_{\chi }}\left(\frac{\partial \vartheta }{\partial \zeta_{j}}\frac{\partial \zeta_{j}}{\partial q_{jk}}\right)\frac{\partial e_{\chi }}{\partial t}\frac{\partial q_{jk}}{\partial t}\\ & =\left[\frac{\partial \vartheta }{\partial q_{jk}}\frac{\partial {\;}^{2}q_{jk}}{\partial t^{2}}\right]+\left[\frac{\partial \zeta_{i}}{\partial t}\left(\frac{\partial^{2}\vartheta }{\partial \zeta_{i}\partial \zeta_{j}}\right)\frac{\partial \zeta_{j}}{\partial t}\right]+\left[\frac{\partial e_{\chi }}{\partial t}\left(\frac{\partial^{2}\vartheta }{\partial e_{\chi }\partial \zeta_{j}}\right)\frac{\partial \zeta_{j}}{\partial t}\right]\\ & =\left[\ddot{\theta }\left(\begin{array}{c} \;c_{s}s_{n}\psi \\ -s_{n}\psi \\ 0 \end{array}\right)+\ddot{\psi }\left(\begin{array}{c} 0\\ 0\\ -1 \end{array}\right)+\ddot{\phi }y_{E}\right]+\left[\dot{\theta }\dot{\psi }\left(\begin{array}{c} -s_{n}\psi \\ -\;c_{s}s_{n}\psi \\ 0 \end{array}\right)\right]+[\dot{\phi }{\dot{y}}_{E}],\\ {\ddot{\vartheta }}_{\left[I\right]} & =\left(\frac{\partial \vartheta_{\left[I\right]}}{\partial q_{jk}}\right)\frac{\partial {\;}^{2}q_{jk}}{\partial t^{2}}+{\ddot{\vartheta }}_{\left[I\right]}^{*},\\ & \;\mathrm{where}\;{\ddot{\vartheta }}_{\left[I\right]}^{*}=\dot{\theta }\dot{\psi }\left(\begin{array}{c} -s_{n}\psi \\ -\;c_{s}s_{n}\psi \\ 0 \end{array}\right)+\dot{\phi }{\dot{y}}_{E}. \end{array}$$

A 13 $$\begin{array}{rl} {\ddot{\vartheta }}_{\left[G\right]} & =\left(\frac{\partial \vartheta_{\left[G\right]}}{\partial q_{jk}}\right)\frac{\partial {\;}^{2}q_{jk}}{\partial t^{2}}+{\ddot{\vartheta }}_{\left[G\right]}^{*}\\ & \;\mathrm{where}\;{\ddot{\vartheta }}_{\left[G\right]}^{*}={\dot{\bar{\Omega }}}_{A[G]}^{*}+{\bar{\Omega }}_{A[G]}\times {\dot{\vartheta }}_{\left[G\right]}+[R_{G,A}]R_{E}{\ddot{\vartheta }}_{\left[I\right]}^{*}. \end{array}$$

Note if ${\bar{\Omega }}_{A[G]}$ is prescribed (i.e. not state-dependent) then ${\dot{\bar{\Omega }}}_{A[G]}^{*}={\dot{\bar{\Omega }}}_{A[G]}$.

A 14 $$\begin{array}{rl} & {\ddot{e}}_{\left[G\right]}=\left(\frac{\partial e_{\left[G\right]}}{\partial q_{jk}}\right)\frac{\partial {\;}^{2}q_{jk}}{\partial t^{2}}+{\ddot{e}}_{\left[G\right]}^{*},\\ & \;\mathrm{where}\;{\ddot{e}}_{\left[G\right]}^{*}={\ddot{\vartheta }}_{\left[G\right]}^{*}\times e_{\left[G\right]}+{\dot{\vartheta }}_{\left[G\right]}\times {\dot{\vartheta }}_{\left[G\right]}\times e_{\left[G\right]}. \end{array}$$

A 15

If ${\bar{r}}_{A[G]}$ is prescribed then ${\ddot{\bar{r}}}_{A[G]}^{*}={\ddot{\bar{r}}}_{A[G]}$.
